# Supplementary figures and images for: Capsular polysaccharide inhibits vaccine-induced O-antigen antibody binding and function across both classical and hypervirulent K2:O1 strains of Klebsiella pneumoniae
Source: PLoS Pathog. 2023 May 5;19(5):e1011367. doi: 10.1371/journal.ppat.1011367 (PMC10191323; doi:10.1371/journal.ppat.1011367)

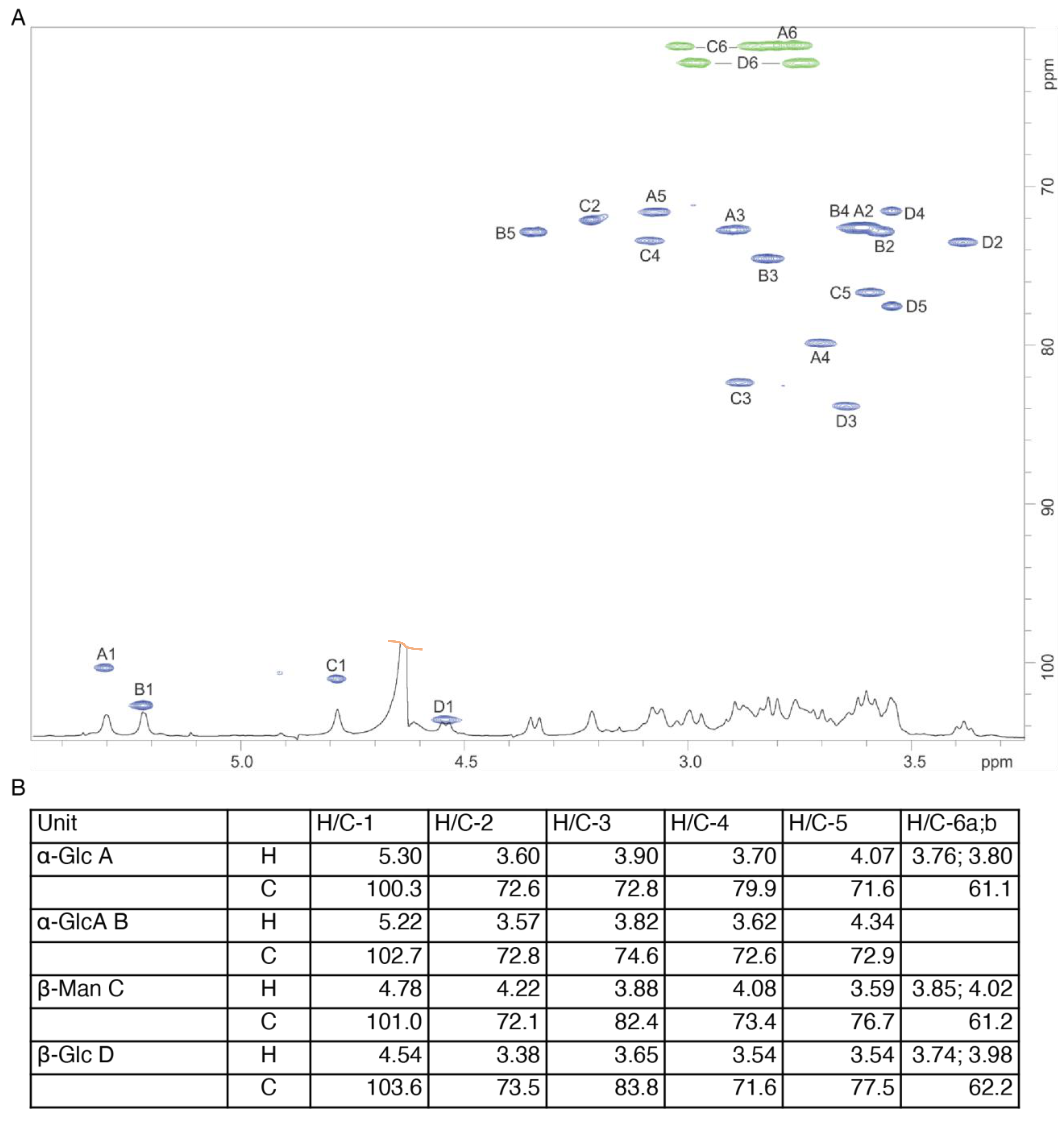

Supplement: S1 Fig — (A) 1H-13C HSQC spectrum of the extracted K2 polysaccharide. Orange hash indicates cut water peak. (B) NMR data for the K2 polysaccharide (D2O, 25°C, 600 MHz). The data show the expected peaks based on the known K2 structure [8]. (TIF) [file ppat.1011367.s001.tif]

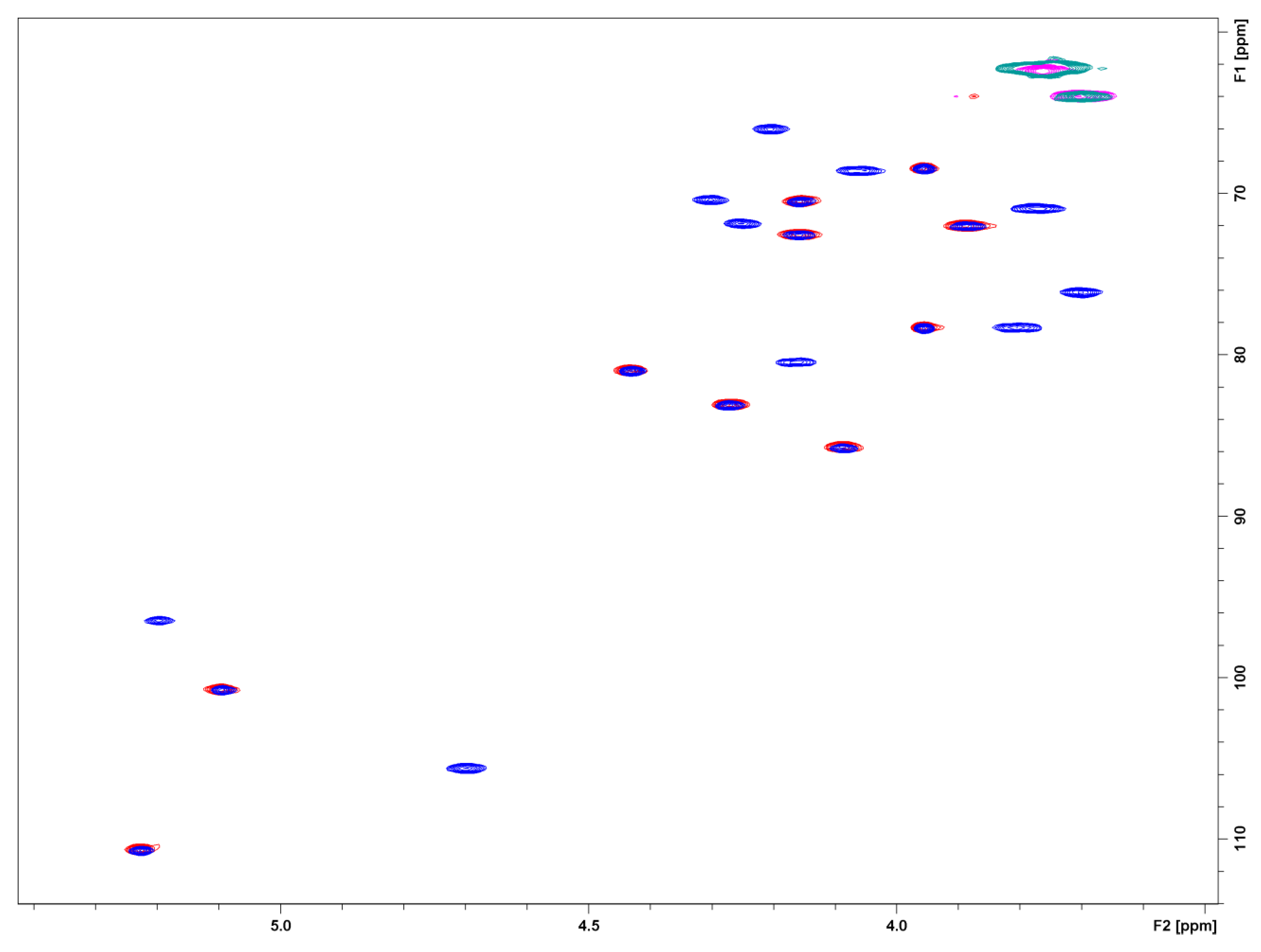

Supplement: S2 Fig — Overlap of the 1H-13C HSQC spectra of Klebsiella O1, galactan II constituent (blue-cyan), and galactan I constituent (red-pink), polysaccharide portions, which is consistent with the known O1 structure [27]. (TIF) [file ppat.1011367.s002.tif]

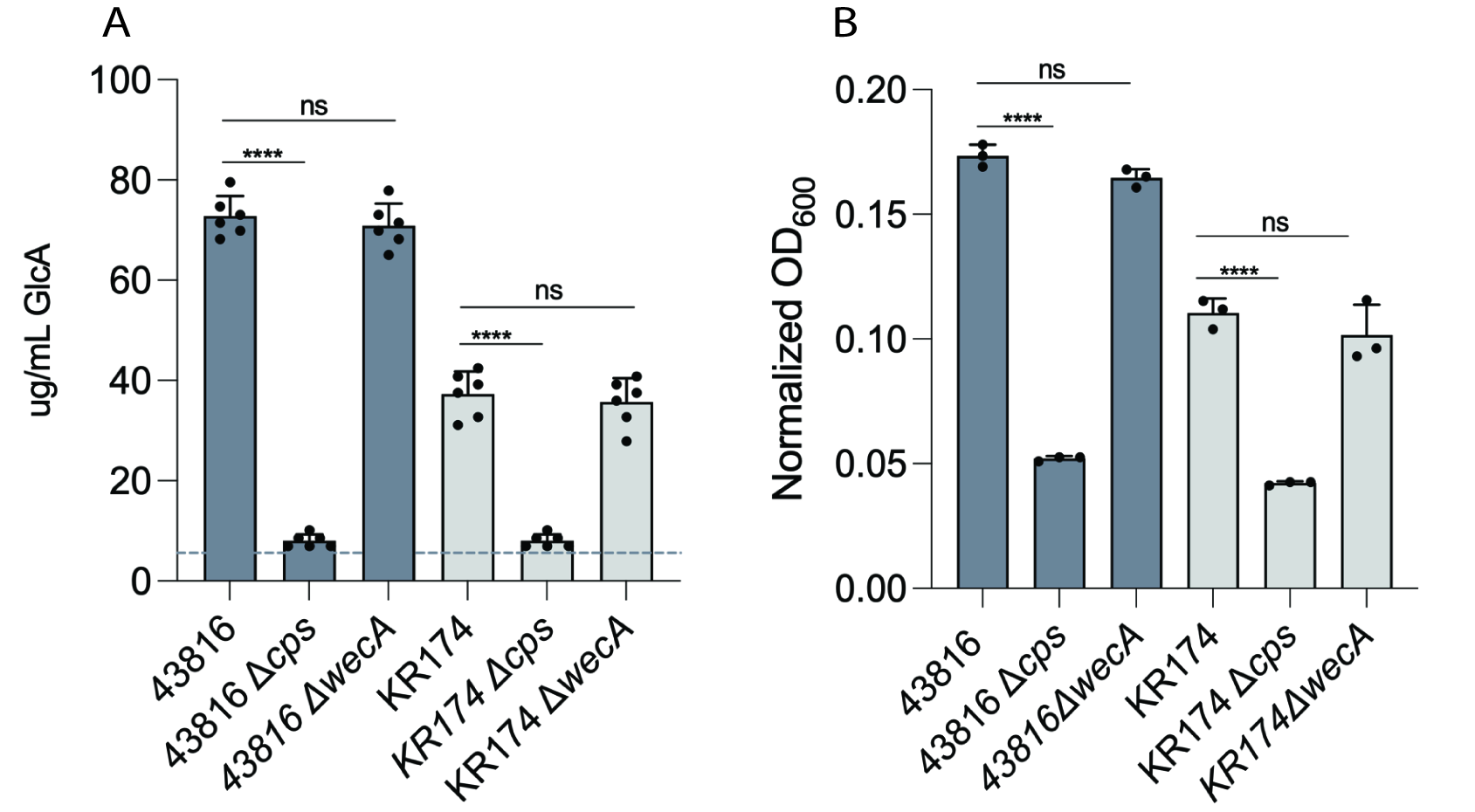

Supplement: S3 Fig — (A) Capsule quantification using a glucuronic acid assay comparing hvKp 43816), cKp KR174, and their respective capsule (cps) and O antigen (wecA) knockouts. The dotted line represents the limit of detection for uronic acid. (B) Hypermucoviscosity quantification via low-speed centrifugation assay. Statistical analyses were performed via Mann-Whitney U test. **** p<0.0001; ns, not significant. (TIF) [file ppat.1011367.s003.tif]

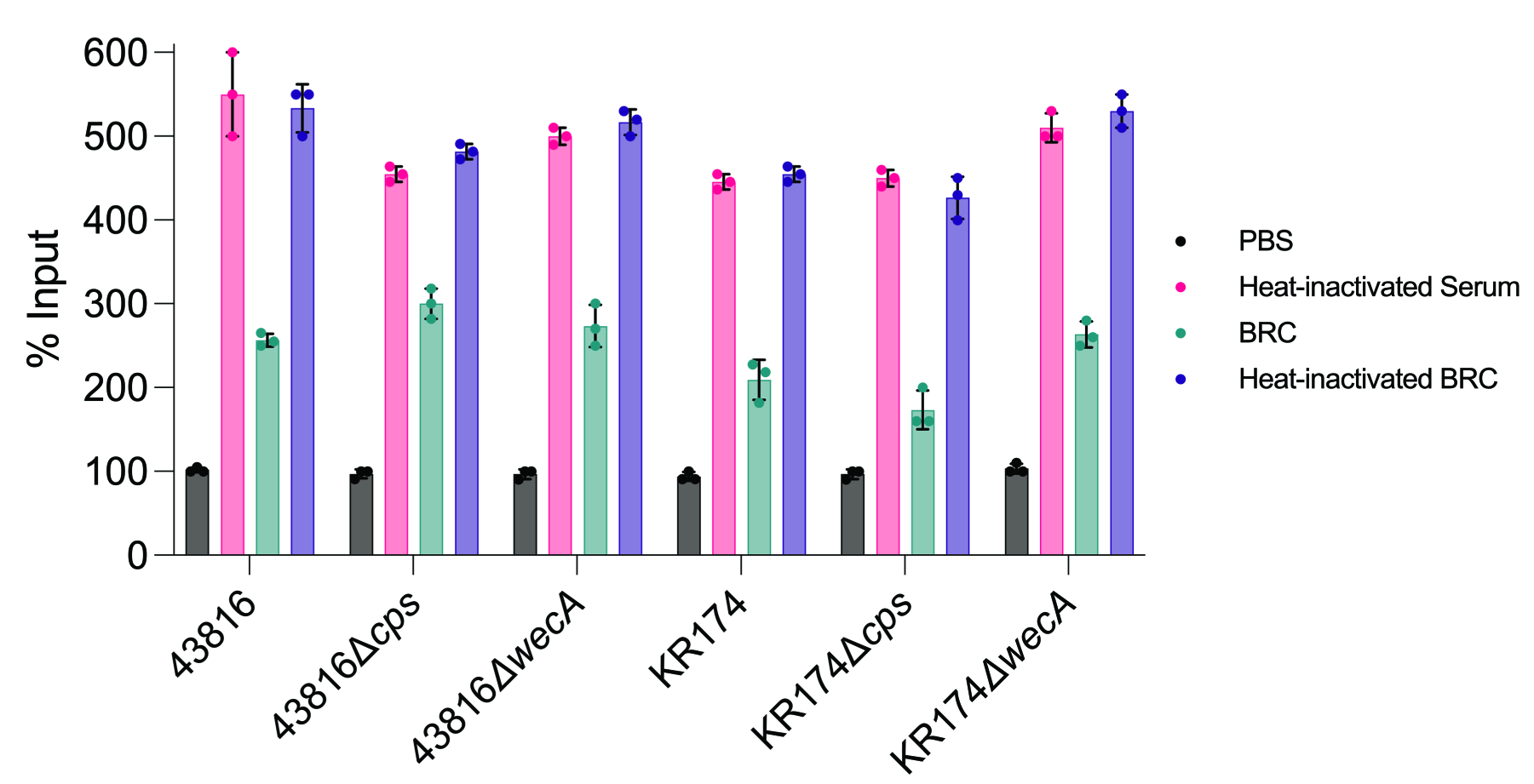

Supplement: S4 Fig — Bacterial strains were incubated in either PBS, heat-inactivated mouse serum, baby rabbit complement (BRC), or heat-inactivated BRC for 2 h at 37°C with shaking prior to plating and enumeration. Counts were determined as percent input relative to the original starting count of each bacterial strain prior to incubation. (TIF) [file ppat.1011367.s004.tif]

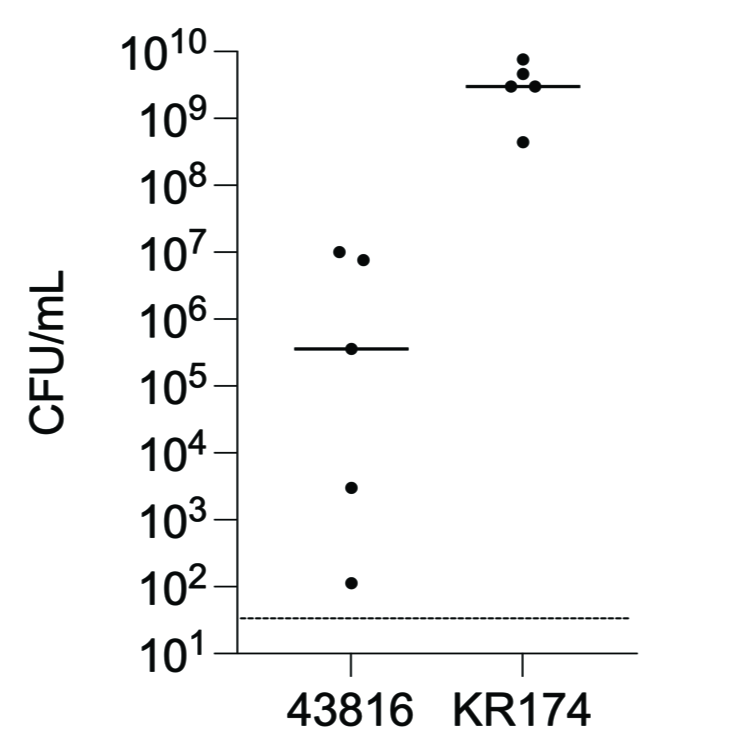

Supplement: S5 Fig — BALB/c mice were infected with 2000 CFU 43816 or 108 CFU KR174 in 50 μL via intraperitoneal injection, and blood was collected for culture after 24 h. The dotted line represents the limit of detection. (TIF) [file ppat.1011367.s005.tif]
