# Supplementary material for: Capsular polysaccharide inhibits vaccine-induced O-antigen antibody binding and function across both classical and hypervirulent K2:O1 strains of Klebsiella pneumoniae
Source: PLoS Pathog. 2023 May 5;19(5):e1011367. doi: 10.1371/journal.ppat.1011367 (PMC10191323; doi:10.1371/journal.ppat.1011367)
Supplement: S1 Table — (DOCX) [file ppat.1011367.s006.docx]

S1 Table: Primers and oligos used in this study.

| Primer | Sequence |
| --- | --- |
| Primers and oligos used to generate pVNM167 | |
| VNM259 | atcatcaccatcatcactaatagagtctgataaaaaatatgtaccatcag |
| VNM260 | aaacagaagcttgcatgcctgcaggtcaatattctattgaacaaaattttaac |
| Primers and oligos used to generate pVNM245 | |
| VNM238 | ctcctcttcaggacccg |
| VNM204 | ccgcgtgaagatttgcatcatcaccatcatcactaatagag |
| VNM237 | gggggtcgtctgacc |
| VNM209 | atgatggtgatgatgcaaatcttcacgcggagg |
| VNM267 | ttacgggtcctgaagaggagtgcaccggcgtgacc |
| VNM268 | agaatggtcagacgacccccgcactgcgcg |
| PCR primers for cloning *K. pneumoniae* O1 O-antigen genes | |
| pBBR1-1F | gcgttaatattttgttaaaattcgcg |
| pBBR1-1R | agctgtttcctgtgtgaaattg |
| O2a_cluster_1F | gcggataacaatttcacacaggaaacagctatgaagtacaatttagggtatttatttg |
| O2a_cluster_1R | ttaacgcgaattttaacaaaatattaacgctcatcgaactacatcatgatatatttg |
| *wbbY*_1F | atgcctgcaggtcgactctattattttaacattgatttcactttccgg |
| *wbbY*_1R | ctcggtacccggggatcctcatgaagaaaattcttataatgacgcc |
| PCR primers for cloning *K. pneumoniae* K2 cluster | |
| pBBR-K2 *wcuF* F | ctaaagggaacaaaagctggtaaaaggataataatgaacgtcatcc |
| pBBR-K2 *wzx* R | ggcatttgagaagcacacggcagcatatttatctctttaagag |
| pWKS-K2 *wcaJ* F | ctaaagggaacaaaagctggtaatgacaatatctcagcatcgc |
| pWKS-K2 *ugd* R | cgacctcgagggggggcccgcagaattaatcgttaccaaacag |
| Primers used to generate *K. pneumoniae* capsule knockout | |
| *wzi* Fwd | aaaatggatcttgtacaatgataaaaattgcgcgcattgccgtgagtgtaggctggagctgcttc |
| *wcaJ* Rvs | atcaataagcagatttgttaataaatcctttaaagacagtaaggaatattcatatgaatatcctccttag |
| *wzi* check | gccgcgagcgctttctatctt |
| *wcaJ* check | ctgcgacacgttcgcagctt |
